# The predictive significance of anti-RO-52 antibody in patients with interstitial pneumonia after treatment of malignant tumors

**DOI:** 10.1515/med-2025-1190

**Published:** 2025-08-04

**Authors:** Zongyu Li, Li Yang, Yan Zhu, Shengli Ye, Xin Fang, Qiuping Xu

**Affiliations:** Department of Respiratory and Critical Care Medicine, Shulan (Hangzhou) Hospital Affiliated to Zhejiang Shuren University Shulan International Medical College, Hangzhou, 330100, China; Department of Respiratory and Critical Care Medicine, The Fourth Affiliated Hospital ZheJiang University School of Medicine, Hangzhou, 322000, China; Department of Radiology, Shulan (Hangzhou) Hospital Affiliated to Zhejiang Shuren University Shulan International Medical College, Hangzhou, 330100, China; Department of Critical Care Medicine, ZheJiang University School of Medicine Sir Run Run Shaw Hospital, No. 3 Qingchun East Road, Shangcheng District, Hangzhou, 310020, China

**Keywords:** malignant tumors, anti-RO-52, interstitial lung disease, predictive

## Abstract

**Aim:**

To discover the predictive indicators of the risk of interstitial lung disease (ILD) after treatment of malignant tumors from myositis-specific autoantibodies (MSA) and myositis-associated autoantibodies (MAA) and explore possible predictive value and significance.

**Methods:**

A total of 73 patients hospitalized in the Shulan (Hangzhou) Hospital from January 2020 to March 2021 were screened retrospectively, they all completed the MSA and MAA, and the imaging was consistent with changes in ILD. We analyzed the characteristics of MSA and MAA in tumor patients and non-tumor patients, and the characteristics of MSA and MAA positive in patients with ILD after treatment.

**Results:**

A total of 58 patients with ILD were diagnosed, 19 patients (32.76%) with malignant tumors, 16 patients with positive MSA or MAA (84.21%), of which 10 (50%) patients had anti-RO-52 antibodies. After treatment, 12 cases (46.15%) developed ILD and 10 cases (90.91%) had a positive spectrum of specific inflammatory diseases.

**Conclusion:**

The MSA and MAA may have a predictive effect on people who are prone to ILD during the treatment of malignant tumors, and the anti-RO-52 antibody may be an important predictive antibody index.

## Graphical abstract

**Figure:**
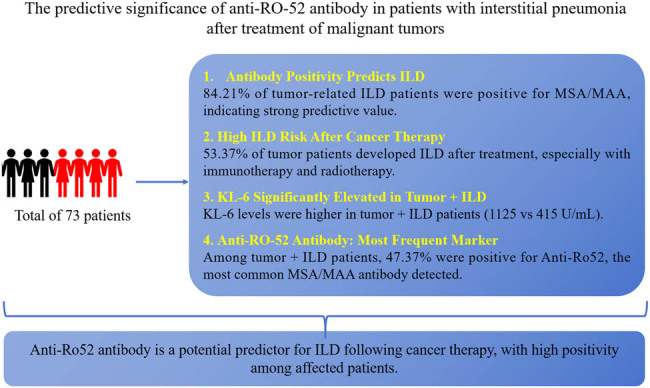

## 1 Introduction

Malignant tumors have now become one of the main public health problems that seriously threaten the health of the Chinese population. According to the latest statistics in 2015, deaths from malignant tumors accounted for 23.91% of all deaths among residents. The mortality rate is second only to heart disease. The current main treatments for tumors are chemotherapy, radiotherapy, targeted therapy, and immunotherapy. It should not be overlooked that during the treatment process with immunosuppressive agents, interstitial lung disease (ILD) has been confirmed as a rare but potentially serious event. Especially for drugs targeting the PD-1/PD-L1 axis, there are even some fatal pneumonia cases [1,2]. At the same time, the occurrence or acute progression of ILD has also been observed in other treatment options for tumors.

ILD is based on diffuse lung parenchyma, alveolar inflammation, and interstitial fibrosis, with active dyspnea as the clinical manifestation. There are many causes of ILD, and currently known causes include environmental factors, pulmonary toxicity drugs, occupational exposure, radiation therapy, and systemic diseases (such as connective tissue diseases, etc.) [3,4]. With the continuous development of cancer treatment methods, tumor-related ILD has gradually become a clinical focus, especially the ILD induced during the treatment of cancer patients. There is an urgent need to further explore its pathogenesis and predictive biomarkers.

In clinical practice, serum myositis-specific autoantibodies (MSA) and myositis-associated autoantibodies (MAA) are widely recognized and used. Each antibody is associated with different clinical phenotypes and outcomes [5–8]. Especially in inflammatory diseases such as myositis, the clinical significance of MSA and MAA has been extensively studied. MSA and MAA are closely related to the immune profile of myositis and provide important clues for the diagnosis, prognosis, and treatment of the disease. In adolescent myositis patients with ILD, those who were positive for anti-Ro-52 antibodies typically experienced a chronic and persistent course, with fewer instances of a single episode. These patients had a longer treatment duration and were more prone to relapse [9]. Additionally, a study evaluating patients with anti-MDA5 antibody-positive, non-myopathic dermatomyositis complicated by pulmonary interstitial disease found that anti-RO-52 antibodies were significantly associated with rapidly progressive ILD and the development of skin ulcers. It was suggested that these patients may have a poor response to conventional immunosuppressive treatment, leading to increased mortality [10]. However, there is no clear report regarding the use of these specific antibody profiles to predict the occurrence of ILD in cancer patients, particularly acute ILD after cancer treatment. To explore the potential role of MSA and MAA in tumor-related ILD, we retrospectively collected data from all patients who underwent MSA or MAA testing (16 items) at the Department of Respiratory and Critical Care Medicine of Shulan (Hangzhou) Hospital between January 1, 2020 and March 1, 2021, and conducted a retrospective analysis. By observing the relationship between MSA or MAA and the occurrence of ILD in cancer patients, particularly the association with ILD after cancer treatment, this study aimed to reveal the clinical value of MSA and MAA as potential biomarkers and further explore their role in reducing the occurrence of ILD in cancer patients.

## 2 Materials and methods

### 2.1 Study design and participants

We conducted a retrospective analysis of all patient records from the Department of Respiratory and Critical Care Medicine of Shulan (Hangzhou) Hospital between January 1, 2020 and March 1, 2021. The inclusion criteria were as follows: (1) imaging findings consistent with ILD changes, including grid shadow, honeycomb sign, ground glass shadow with traction bronchiectasis, peripheral or bronchial consolidation ground glass shadow, distribution around lobules, central lobular nodules with unclear borders, and subpleural line shadows; (2) completion of comprehensive MSA and MAA testing (referred to as the myositis panel), including anti-JO-1 antibodies, anti-PL-7 antibodies, anti-PL-12 antibodies, anti-EJ antibodies, anti-OJ antibodies, anti-SRPα antibodies, anti-HMGCR antibodies, anti-cN-1A antibodies, anti-Mi-2 antibodies, anti-Mi-2α antibodies, anti-Mi-2β antibodies, anti-NXP-2 antibodies, anti-SAE antibodies, anti-TIF1-Y antibodies, anti-MDA5 antibodies, anti-Ku antibodies, anti-PM-Scl100 antibodies, anti-PM-Scl75 antibodies, anti-Ro-52 antibodies, and anti-U1-nRNP antibodies; and (3) patients aged over 18 years. The exclusion criteria were (1) patients with lung involvement due to drug or environmental exposure history, (2) pregnant or breastfeeding women, and (3) patients lacking relevant clinical data. A total of 773 patients were included in the final analysis.

### 2.2 Diagnostic criteria

Once all selected patients had changes in ILD found on chest imaging, based on the patient’s medical history, physical examination, laboratory tests, and high-resolution CT (HRCT) scans, the patients were divided into ILD and non-ILD. Then, based on imaging, ILD patients were divided into two categories: usual interstitial pneumonia (UIP) and non-UIP. All patient data and imaging were reviewed by clinical experts and imaging experts with experience in ILD in our hospital. Idiopathic interstitial pneumonia (IIP) diagnostic criteria referred to the classification opinions and diagnostic criteria of IIPs published by the American Thoracic Society and European Respiratory Society in 2013 [11].

### 2.3 Data collection

Data from all patients were collected through the hospital’s electronic medical record system, covering the period from January 1, 2020 to March 1, 2021. We collected basic information, including gender, age, history, time of onset (from the onset of symptoms to diagnosis), tumor type, and imaging typing; 22 items of autoantibodies, anti-myeloperoxidase antibodies (MPO), antiproteinase 3 antibody (PR3), anti-streptolysin O (ASO), anti-cyclic citrullinated peptide antibody (CCP), C-reactive protein (CRP), rheumatoid factor (RF), sialic sugar chain antigen KL-6 detection (KL-6), erythrocyte sedimentation rate (ESR), myositis spectrum, and other serologic tests; as well as imaging assessments, including chest X-ray, CT, and HRCT.

The rheumatic and autoimmune diseases addressed in this study include: dermatomyositis, characterized by skin damage and muscle weakness, with classification criteria based on recent research [12]; rheumatoid arthritis (RA) is a chronic, systemic autoimmune disease primarily affecting the joints and may also involve other organs, and relevant classification criteria for RA can be found in pertinent reports [13]; systemic sclerosis is defined by fibrosis of the skin and internal organs, with clearly established diagnostic criteria [14]; Sjögren’s syndrome primarily manifests as dry eyes and dry mouth, potentially accompanied by other systemic symptoms, with classification standards derived from the latest reports [15].

### 2.4 Detection method of idiopathic inflammatory myopathy spectrum antibody

Serum from all patients was sent to the Hangzhou European Union Medical Laboratory for testing, using the anti-myositis antibody profile 4 IgG detection kit (EUROLine western blot assay) and EUROBlotMaster II fully automatic immunoblotting and interpretation system for testing. Operation steps: After centrifugation, 15 μL of the test serum was diluted and mixed with 1.5 mL of sample buffer. The test strip, with the numbered side facing up, was placed into the incubation trough. Each trough was filled with 1.5 mL of sample buffer and incubated for 5 minutes. The buffer was then removed, and the diluted serum samples were added to the troughs and incubated on a shaker for 30 minutes. Next, 1.5 mL of wash buffer was used to wash the strips three times, each for 5 minutes. Then, 1.5 mL of alkaline phosphatase-labeled anti-human IgG was added and incubated on a shaker for 30 minutes, followed by another round of washing using wash buffer. Subsequently, 1.5 mL of substrate solution was added to each trough for color development for 10 minutes. The strips were then rinsed three times with distilled water, each for 1 minute. Finally, the test strips were placed on the interpretation template and allowed to air dry. The results were evaluated using EUROLineScan software.

### 2.5 Statistical methods

SPSS 26.0 software was used for statistical analysis. Normally distributed continuous data were expressed as mean ± standard deviation, and a *t*-test was used for comparison between groups. Non-normally distributed continuous data were expressed as median (range), and the Mann–Whitney *U-*test was used for comparison between groups. Categorical data are expressed as *n* (%), with group comparisons made using the Chi-square test.


**Ethics approval:** This study is approved by the Ethics Committee of Shulan (Hangzhou) Hospital (Reference Number: KY2023051).

## 3 Results

### 3.1 General conditions of all selected patients

We analyzed 73 patients, including 35 males (47.95%) and 38 females (52.05%). Among them, 58 patients were diagnosed with ILD.

### 3.2 Analysis of the general conditions of patients diagnosed with ILD

Among the 58 patients diagnosed with ILD, 19 were identified as malignant tumor cases (32.76%) and 39 were classified as non-tumor cases (67.24%). The cohort consisted of 31 male patients and 27 female patients. Imaging examinations revealed 28 cases (48.28%) of non-UIP and 30 cases (51.72%) of UIP. There was no statistically significant difference in the mean age and gender of the patients in both groups (Table 1). However, the prevalence of rheumatic immune diseases was significantly higher in non-tumor patients (*P* < 0.05), specifically including dermatomyositis (five cases), RA (two cases), scleroderma (one case), and Sjögren’s syndrome (one case). The tumor group had significantly higher levels of CRP compared to the non-tumor group (57.4 vs 17.6, *P* < 0.05).

**Table 1:** Baseline characteristics of the two groups of patients

Index characteristics of ILD	Tumor patients (*N* = 19)	Non-tumor patients (*N* = 39)	*P*
**Basic situation**			
Age	63.05 (34–88)	68.15 (48–92)	0.129
Gender, male (*n*, %)	12 (63.2)	19 (48.7)	0.309
**Past history**			
Rheumatic immune disease (*n*, %)	0 (0)	9 (23.1)	0.023
Structural lung disease (*n*, %)	1 (5.3)	3 (7.7)	0.737
Diabetes (*n*, %)	5 (26.3)	8 (20.5)	0.662
**Laboratory diagnosis**			
KL-6	1125.2 (361–3309)	1290.8 (232–4732)	0.535
ESR	52.7 (8–104)	41.3 (7–121)	0.211
CRP	57.4 (2.2–182.8)	17.6 (0.5–99.8)	0.001
MPO	6.7 (0.1–64.2)	17.2 (0.1–201.8)	0.403
PR3	20.2 (0.5–185.7)	10.2 (0.1–92.9)	0.379
CCP	4.4 (0.5–52.4)	8.2 (0.5–250)	0.717
ASO	48.8 (4.7–130.2)	40.5 (6.2–121)	0.437
RF	21 (3.2–76.8)	18.4 (2.7–194.9)	0.771
**22 autoantibodies**			
Antinuclear antibody (*n*, %)	6/18 (33.3)	31/37 (83.8)	0.0001
Antineutrophil perinucleus antibody (*n*, %)	1/18 (5.6)	8/37 (21.6)	0.166
Anti-SSA antibody (*n*, %)	0/18 (0)	5/37 (13.5)	0.121
Anti-mitochondrial antibody type 2 antibody IgG (*n*, %)	1/18 (5.6)	1/37 (2.7)	0.56
Anti-ScL-70 antibody (*n*, %)	0/18 (0)	2/37 (5.4)	0.343
**MSA and MAA**			
Anti-JO-1 antibody IgG (*n*, %)	2/19 (10.5)	2/39 (5.1)	0.472
Anti-MDA5 antibody IgG (*n*, %)	1/19 (5.26)	0/39 (0)	0.159
Anti-RO-52 antibody (*n*, %)	9/19 (47.4)	11/39 (28.2)	0.129
Anti-Mi-2α antibody IgG (*n*, %)	2/19 (10.5)	0/39 (0)	0.042
Anti-PL-7 antibody IgG (*n*, %)	1/19 (5.26)	7/39 (17.9)	0.184
Anti-NXP2 antibody IgG (*n*, %)	1/19 (5.26)	0/39 (0)	0.159
Anti-EJ antibody IgG (*n*, %)	1/19 (5.26)	2/39 (5.1)	0.983
Anti-Mi-2β antibody IgG (*n*, %)	1/19 (5.26)	2/39 (5.1)	0.983
Anti-SAE1 antibody IgG (*n*, %)	1/19 (5.26)	1/39 (2.56)	0.618
Anti-PM-Scl75 antibody IgG (*n*, %)	1/19 (5.26)	0/39 (0)	0.159
Anti-TIF1γ antibody IgG (*n*, %)	1/19 (5.26)	1/39 (2.56)	0.618
Anti-PL-12 antibody IgG (*n*, %)	0/19 (0)	2/39 (5.1)	0.317
Anti-PM-Scl100 antibody IgG (*n*, %)	0/19 (5.26)	3/39 (7.69)	0.215
Anti-SRP antibody IgG (*n*, %)	0/19 (0)	3/39 (7.69)	0.215
Anti-Ku antibody IgG (*n*, %)	0/19 (0)	1/39 (2.56)	0.484
**Radiology**			
UIP (*n*, %)	8/19 (42.1)	22/39 (56.4)	0.315
Non-UIP (*n*, %)	11/19 (57.9)	17/39 (43.6)	0.315

We compared and analyzed the myositis spectrum in both groups of patients. Among them, anti-Mi-2α antibody IgG (2 vs 0, *P* < 0.05) was present only in cancer patients; however, due to the small sample size, this finding was not considered statistically significant. In other myositis spectrums, we observed the highest incidence of anti-RO-52 antibodies, totaling 20 cases. Among non-tumor and tumor patients, there were 11 (28.2%) and 9 (47.4%) cases, respectively. The results showed that anti-RO-52 antibodies were present in a higher percentage of cancer patients.

### 3.3 Situation of patients with tumors complicated by imaging changes in lung interstitial pneumonia

#### 3.3.1 General characteristics of patients with tumors and ILD

To determine whether the occurrence of ILD in tumor patients is associated with a specific myositis spectrum, we re-analyzed the tumor patients, with results shown in Table 2. Among the 27 tumor patients, 19 had ILD and 8 had non-ILD. In the ILD group, there were 12 male and 7 female patients, with an age range of 63.1 (34–88) years. Six cases had UIP and 13 had non-UIP. Among the 27 tumor patients, lung cancer was the most common (twelve cases), followed by breast cancer (six cases) and colorectal cancer (three cases). ILD occurred in 15 of the tumor patients after treatment (53.37%), with 3 cases following radiotherapy, 4 cases each after surgery and immunotherapy, and 1 case each after targeted therapy, endocrine therapy, targeted combined chemotherapy, and radiotherapy combined chemotherapy. Except for gender, there were no statistically significant differences in the basic characteristics between the two groups (*P* > 0.05).

**Table 2:** Baseline characteristics of tumor patients

Characteristic	Interstitial pneumonia (*n* = 19)	Non-interstitial pneumonia (*n* = 8)	*P*
**Gender**			0.033
Male	12 (63.16)	1 (12.50)	
Female	7 (36.84)	7 (87.50)	
Age	63.1 (34–88)	64.63 (47–82)	0.775
**Tumor type**			0.483
Lung cancer	9 (47.37)	3 (37.50)	
Breast cancer	5 (26.32)	1 (12.50)	
Colorectal cancer	1 (5.26)	2 (25.00)	
Others	4 (21.05)	2 (25.00)	
**Stage**			0.078
I–II	2 (10.53)	4 (50.00)	
III–IV	10 (52.63)	3 (37.50)	
Unknown	7 (36.84)	1 (12.50)	
**Treatment history before onset**			0.152
None	4 (21.05)	1 (12.50)	
Surgery	4 (21.05)	3 (37.50)	
Chemotherapy	0	2 (25.00)	
Radiotherapy	3 (15.79)	0	
Targeted therapy	1 (5.26)	0	
Immunotherapy	4 (21.05)	1 (12.50)	
Endocrine therapy	1 (5.26)	0	
Radiotherapy + chemotherapy	1 (5.26)	0	
Targeted + chemotherapy	1 (5.26)	0	
Immunotherapy + radiotherapy	0	1 (12.50)	
**Type of ILD**			—
UIP	6/19 (31.58)	—	
Non-UIP	13/19 (68.42)	—	

Others include esophageal cancer, pharyngeal cancer, cholangiocarcinoma, bladder cancer, and kidney cancer.

When categorized by tumor type, the results indicated a relative higher incidence of ILD in breast cancer patients (83.33%), all of whom presented with the non-UIP type. Additionally, a higher proportion of ILD was noted in stage III–IV lung cancer patients, suggesting a potential correlation between disease progression and pulmonary complications (Table 3).

**Table 3:** Status of interstitial pneumonia in different tumor patients

Characteristic	Lung cancer (*n* = 12)	Breast cancer (*n* = 6)	Colorectal cancer (*n* = 3)	Others (*n* = 6)	*P*
**Gender**					0.014
Male	8 (66.67)	—	1 (33.33)	4 (66.67)	
Female	4 (33.33)	6 (100.00)	2 (66.67)	2 (33.33)	
Age	64.8 (34–88)	57.2 (47–70)	62.7 (47–75)	65.5 (57–82)	0.572
**Stage**					0.088
I–II	3 (25.00)	1 (16.67)	1 (33.33)	1 (16.67)	
III–IV	5 (41.67)	5 (83.33)	2 (66.67)	1 (16.67)	
Unknown	4 (33.33)	0	0	4 (66.67)	
**Treatment history before onset**					0.190
None	4 (33.33)	0	0	1 (16.67)	
Surgery	4 (33.33)	0	1 (33.33)	2 (33.33)	
Chemotherapy	0	1 (16.67)	1 (33.33)	0	
Radiotherapy	2 (16.67)	0	0	1 (16.67)	
Targeted therapy	0	1 (16.67)	0	0	
Immunotherapy	2 (16.67)	2 (40.00)	0	1 (16.67)	
Endocrine therapy	0	1 (16.67)	0	0	
Radiotherapy + chemotherapy	0	1 (16.67)	0	0	
Targeted + chemotherapy	0	0	1 (33.33)	0	
Immunotherapy + radiotherapy	0	0	0	1 (16.67)	
**Is ILD**					0.483
Yes	9 (75.00)	5 (83.33)	1 (33.33)	4 (66.67)	
No	3 (25.00)	1 (16.67)	2 (66.67)	2 (33.33)	
**Type of ILD**					0.122
UIP	4/9 (44.44)	0/5	0/1	2/4 (50.00)	
Non-UIP	5/9 (55.56)	5/5 (100.00)	1/1 (100.00)	2/4 (50.00)	

Others include esophageal cancer, pharyngeal cancer, cholangiocarcinoma, bladder cancer, and kidney cancer.

#### 3.3.2 Analysis of laboratory indices in patients with tumors complicated by interstitial pneumonia

The average value for patients with malignant tumors complicated by interstitial pneumonia was significantly higher than that for patients with malignant tumors without interstitial pneumonia (1,125 U/mL vs 415 U/mL, *P* < 0.05). However, the ESR between the two groups (42.7 vs 52.7, *P* > 0.05) was not statistically significant (Table 4). All patients underwent tests for 22 types of serum autoantibodies. ANCA titer ≥ 1:100 was considered positive. In the group with malignant tumors and interstitial pneumonia, the positive rate of ANCA was 31.58% (six cases). In the group of malignant tumors without ILD, the positive rate of ANCA was 50% (four cases). There was no statistically significant difference in the ANCA positive rates between the two groups (*P* > 0.05).

**Table 4:** Laboratory diagnosis and 22 autoantibodies of the two groups

Index characteristics of ILD	Tumor with interstitial pneumonia patient group (*N* = 19)	Tumor with non-interstitial pneumonia group (*N* = 8)	*P*
**Laboratory diagnosis**			
KL-6	1,125 (361–3,309)	415 (197–480)	0.042
MPO	6.7 (0.1–64.2)	1.1 (0.1–4.2)	0.578
PR3	20.2 (0.5–185.7)	6.8 (3.5–14.6)	0.642
CCP	4.6 (0.5–42.6)	0.7 (0.5–1.1)	0.497
CRP	60.7 (2.2–182)	33.5 (1.7–102)	0.369
ESR	52.7 (8–104)	42.7 (8–87)	0.411
RF	22 (3.2–76.8)	14.7 (8.6–38.7)	0.515
ASO	4,844 (4.7–130.2)	28.1 (12.3–45.4)	0.292
**22 autoantibodies**			
ANCA	6	4	0.345
Antineutrophil perinucleus antibody	1	0	0.533
Antineutrophil cytoplasmic antibody	0	1	0.121
Ribosomal P protein antibody	0	1	0.121
Anti-mitochondrial antibody type 2 antibody IgG	1	0	0.533
Anti-ScL-70 antibody	0	1	0.121
**MAS and MAA**			
Anti-JO-1 antibody IgG	2	0	0.392
Anti-MDA5 antibody IgG	1	0	0.555
Anti-RO-52 antibody	9	0	0.024
Anti-Mi-2α antibody IgG	2	0	0.392
Anti-PL-7 antibody IgG	1	0	0.555
Anti-NXP2 antibody IgG	1	0	0.555
Anti-EJ antibody IgG	1	0	
Anti-Mi-2β antibody IgG	1	0	0.392
Anti-SAE1 antibody IgG	1	0	0.555
Anti-PM-Scl75 antibody IgG	1	0	0.555
Anti-TIF1γ antibody IgG	1	0	0.555
Anti-PL-12 antibody IgG	0	0	
Anti-PM-Scl100 antibody IgG	0	0	
Anti-Ku antibody IgG	0	0	
Anti-EJ antibody IgG	0	0	
Anti-PM-Scl100 antibody IgG	0	0	
Anti-SRP antibody IgG	1	0	0.555

#### 3.3.3 Predictive value of 16 items in the spectrum of idiopathic inflammatory myopathy in patients with tumors and interstitial inflammation

We tested the myositis spectrum for all enrolled patients. Of the 19 enrolled patients with tumors and interstitial pneumonia, 16 exhibited positive antibody profiles (84.21%) and 3 cases showed no positive antibody profiles (15.79%). Among these, nine patients tested positive for anti-RO-52 antibody; two cases each were positive for anti-JO-1 antibody IgG and anti-Mi-2α antibody IgG; and one case each was positive for anti-Mi-2β antibody IgG, anti-MDA5 antibody IgG, anti-PL-7 antibody IgG, anti-NXP2 antibody IgG, anti-EJ antibody IgG, anti-PM-Scl75 antibody IgG, and anti-TIF1γ antibody IgG; other antibodies (such as anti-ANA, anti-Sm, anti-dsDNA, anti-RNP, anti-Scl-70, and anti-ACA antibody IgG) were not detected. Notably, anti-RO-52 positivity was most frequently observed in patients with tumors and interstitial pneumonia (47.37%), and the positive rate among patients with interstitial pneumonia following treatment reached 73.73% (Table 4).

In addition, we observed that 15 of the 19 patients with tumors and interstitial pneumonia received tumor-related treatments, of which 13 cases (86.67%) were positive for the spectrum of specific inflammatory diseases. Four tumor patients did not receive tumor-related treatment for various reasons and three cases (75%) showed a positive myositis spectrum. Among patients with tumors without interstitial pneumonia, all eight patients underwent cancer treatment, and none exhibited positivity for a specific inflammatory disease spectrum.

## 4 Discussion

In this study, we found that compared with tumor patients who did not develop interstitial pneumonia, the positive rate of myositis spectrum in tumor patients who developed ILD after treatment was significantly higher. In addition, patients with positive myositis spectrum had a significantly shorter time for interstitial pneumonia after tumor treatment.

At present and in the future, to reduce the adverse reactions of tumor patients after treatment, everyone has gradually paid attention to it. We reviewed previous literature studies and found that there have been related reports on the relationship between tumors and ILD, especially between lung cancer and ILD, considering that the two have common risk factors, such as smoking or exposure to chemicals [16,17]. The pathophysiology of ILD is similar to the development process of cancer, including epithelial cell abnormalities, bioenergetics from metaplasia to cancerous cells, soluble media release, genetic changes, and telomere wear that lead to aging and abnormal development pathways [18].

The current treatment methods for tumors are mostly surgery, radiotherapy and chemotherapy, immune and targeted therapy, and the occurrence of interstitial pneumonia or the acute progress of the original interstitial pneumonia can be observed in these treatment methods [19,20], there are also non-A case report of ILD after lung cancer immunotherapy and radiotherapy [19], Kobayashi et al. [21] retrospectively analyzed ILD patients receiving locally advanced non-small cell lung cancer (LA-NSCLC) radiotherapy and chemotherapy. Among 37 patients, 17 patients (46%) had acute exacerbations of ILD greater than or equal to grade 3 within 1 year after radiotherapy. The incidence of acute exacerbations of ILD in non-UIP patients was lower than that in UIP patients. There are also reports of severe pulmonary toxicity and even death for immunotherapy, especially drugs targeting the PD-1/PD-L1 axis [1,2,20]. The diagnosis of immune-related interstitial pneumonia (ICI-ILD) is not straightforward. Clinicians should remain highly skeptical if new respiratory symptoms appear while receiving ICI. As we all know, most of these ILD cases occur in the first few months of treatment (median time to onset is 2.3 months) [22,23]. In a meta-analysis, the incidence of ILD in NSCLC was significantly higher, providing evidence for this hypothesis [24]. Targeted therapy, such as ErbB receptor tyrosine kinase inhibitor (EGFR-TKI), although the incidence of ILD caused by EGFR-TKI is not high, once it occurs, it can seriously threaten the life of patients [25]. There are also related reports and studies on chemotherapy; for instance, bleomycin can cause pulmonary fibrosis [26].

The spectrum of specific inflammatory diseases includes serum MSA and serum MAA, which are the diagnostic markers of many systemic autoimmune rheumatic diseases. More and more related articles have been published and confirmed that it has predictive significance for the severity and progression of the disease and tumors in idiopathic inflammatory myopathy-related ILD [27]. Many studies have stated that idiopathic pulmonary fibrosis (IPF) and lung cancer have common risk factors, such as smoking or exposure to chemicals [16,17]. The positive rate of myositis spectrum in patients with qualitative pneumonia accounted for 84.21%, and the positive rate of myositis spectrum in patients after tumor treatment reached 90.91%. Therefore, we believe the spectrum of specific inflammatory diseases is important for ILD after tumor treatment. Occurrence has certain predictive significance.

Acute interstitial pneumonia (AIP) is a rapid progressive ILD whose pathological features are extensive pulmonary septal edema and type I and type II lung cell desquamation. Now, more and more pieces of literature focus on the acute exacerbation of ILD other than IPF [28].

In this article, we observed that anti-RO-52 antibody-positive patients were common in patients with tumors with interstitial pneumonia. The positive rate of patients with tumors with interstitial pneumonia accounted for 47.37%. For the antibody myositis spectrum in patients after tumor treatment, the positive rate reached 72.73%, and we compared patients with interstitial pneumonia anti-RO-52 antibody positive after tumor treatment and patients with untreated interstitial pneumonia, compared with the average lung time from symptom onset to diagnosis. Based on the above observations, we hypothesize that anti-RO-52 antibodies have a certain predictive value in patients with AIP after tumor treatment, but our clinical data are limited, and more data are needed to verify later.

KL-6 is a mucin-like molecule expressed in type II lung cells and respiratory bronchiolar epithelial cells in normal lungs [29]. KL-6 levels reflect the condition of parenchymal cells in lung tissue surrounding tissue damage and can serve as a serum biomarker for the diagnosis and monitoring of ILD. In this article, we observed that KL-6 in patients with interstitial pneumonia was significantly higher than that in patients with non-interstitial pneumonia. A study by Kohnet and others considers that in the context of interstitial pneumonia, the elevation of KL-6 in the patient’s serum is caused by damaged or regenerated epithelial cells in the lower respiratory tract. Similar results have also been reported in lung cancer patients who developed radiation pneumonitis [29]. Therefore, we can monitor the KL-6 value to predict the occurrence of radiation pneumonitis in patients after tumor treatment, especially after radiotherapy.

However, there are certain limitations in this study. First, monitoring KL-6 levels may provide some indication of ILD development, but due to our small sample size, statistical errors may exist. Second, although we preliminarily observed that CRP levels may be related to cancer prognosis, different types of cancer are associated with varying degrees of inflammatory response. However, since this study is a retrospective analysis with a limited sample size, we cannot draw universally applicable conclusions. Finally, due to the retrospective design of this study, detailed clinical features of some patients (such as muscle enzyme levels, skin changes, etc.) were not adequately collected, so we cannot determine whether they meet the classification criteria for immune-mediated myopathy. This missing data limits our comprehensive assessment of the patient’s condition. Future large-scale, prospective studies are needed to verify the potential of KL-6 as an early biomarker for ILD and further explore the relationship between CRP levels and cancer prognosis. We also recommend conducting long-term follow-up studies to assess the predictive role of anti-Ro-52 antibody positivity in cancer treatment-related AIP. Additionally, more clinical data, particularly muscle enzyme levels and skin changes, should be collected to more accurately evaluate whether cancer patients meet the diagnostic criteria for immune-mediated myopathy, thus providing more comprehensive guidance for clinical treatment.

In conclusion, the mechanism of ILD combined with interstitial pneumonia in cancer treatment is still unclear, but we speculate that it may involve the disruption of pulmonary interstitial T cells, triggering an immune response. We believe that specific inflammatory disease profiles may have a predictive role in cancer patients prone to interstitial pneumonia, with anti-RO-52 antibody positivity potentially associated with an increased risk of AIP after cancer treatment. This finding may provide a reference for patient care during cancer treatment.
